# Alterations in magnetic resonance imaging characteristics of bioabsorbable magnesium screws over time in humans: a retrospective single center study

**DOI:** 10.1515/iss-2021-0032

**Published:** 2021-12-23

**Authors:** Lena Sonnow, Andreas Ziegler, Gesa H. Pöhler, Martin H. Kirschner, Maximilian Richter, Mustafa Cetin, Melih Unal, Ozkan Kose

**Affiliations:** Department of Diagnostic and Interventional Radiology, Hannover Medical School, Hannover, Germany; Medizincampus Davos, Davos, Switzerland; School of Mathematics, Statistics and Computer Science, University of KwaZulu-Natal, Pietermaritzburg, South Africa; Department of Cardiology, University Heart & Vascular Center Hamburg, University Medical Center Hamburg-Eppendorf, Hamburg, Germany; Syntellix AG, Hannover, Germany; Private Practice Center Leinhausen, Hannover, Germany; Department of Radiology, Antalya Education and Research Hospital, Antalya, Turkey; Department of Orthopedics and Traumatology, Antalya Education and Research Hospital, Antalya, Turkey

**Keywords:** bioabsorbable implants, magnesium (Mg) implants, magnetic resonance imaging (MRI), metal artifacts, orthopedic implants

## Abstract

**Objectives:**

This study aimed to examine the alterations in magnetic resonance imaging (MRI) characteristics of bioabsorbable magnesium (Mg) screws over time in a single center study in humans.

**Methods:**

Seventeen patients who underwent medial malleolar (MM) fracture or osteotomy fixation using bioabsorbable Mg screws and had at least one postoperative MRI were included in this retrospective study. Six of them had more than one MRI in the postoperative period and were subject of the artifact reduction measurements. 1.5T or 3T MRI scans were acquired in different periods in each patient. The size and extent of the artifact were assessed independently by two experienced radiologists both quantitatively (distance measurement) and qualitatively (Likert scale).

**Results:**

In the quantitative measurements of the six follow-up patients the screw’s signal loss artifact extent significantly decreased over the time, regardless of the MRI field strength (p<0.001). The mean artifact reduction was 0.06 mm (95% confidence interval [CI]: 0.05–0.07) for proton density weighted [PDw] and 0.04 mm (95% CI: 0.03–0.05) for T1 weighted (T1w) sequences per week. The qualitative assessments similarly showed significant artifact reduction in all MRI sequences. Different imaging findings, like bone marrow edema (BME), liquid collections, and gas formation were reported. The overall inter-reader agreement was high (κ=0.88, p<0.001).

**Conclusions:**

The time-dependent artifact reduction of Mg screws in postoperative controls might indicate the expected self-degradation of the Mg implants. In addition, different MRI findings were reported, which are characteristic of Mg implants. Further MRI studies are required to get a better understanding of Mg imaging properties.

## Introduction

Medial malleolar (MM) screw fixation is usually required in two common clinical indications. The first indication is the displaced MM fracture, which is one of the most common fractures in adults with an incidence of up to 174/100,000 persons/year [1, 2]. The second indication is the MM osteotomy, a well-established surgical approach to extend the ankle joint exposure to treat osteochondral lesions of the talus (OLT) [3, 4]. In the treatment of both conditions, restoration of ankle stability, anatomic reduction, and achieving bone union are fundamental surgical goals that require reliable orthopedic implants, such as titanium or stainless-steel screws. Although the indications for routine implant removal is still controversial, implant removal is a major clinical issue with these permanent metallic implants [5]. Soft tissue coverage is poor and subcutaneous fat around the medial malleolus is not bulky, and thus implant-related pain, skin irritation, and difficulties in shoe wearing frequently occur following ankle fractures. Thus, the ankle is the most common anatomical location, where implant removal operations are carried out [6]. Implant removal was required in up to 31% after ankle fractures and in 71% after MM osteotomies [7, 8]. The possible benefits of implant removal should be carefully weighed against the risk of complications like soft tissue damage or infection as well as the increase of costs due to a second operation [5].

Consequently, biodegradable materials have gained importance in ankle surgery. Bioabsorbable implants made of materials such as synthetic polymers were developed over the years and showed similar results as metallic implants [9], However complications such as local foreign-body reactions occurred [10, 11]. Biodegradable screws made of magnesium (Mg) alloys with more than 90% Mg were introduced as an alternative orthopedic implant [12]. Previous studies showed comparable therapeutic effects of Mg compression screws compared to titanium screws in hallux valgus surgery [13], [14], [15], [16]. Recent studies demonstrated a similar functional and radiological outcome of bioabsorbable Mg compared to titanium screws for MM osteotomy fixation in treating OLT as well as MM fracture fixation [17, 18].

In imaging Mg screws have advantageous properties compared to other metallic implants. In conventional radiography, Mg screws show a reduced attenuation of X-rays compared to titanium screws. In computed tomography (CT) and magnetic resonance imaging (MRI), Mg screws generate substantially fewer artifacts compared to titanium screws and may therefore facilitate postoperative imaging follow-up [19, 20]. Especially in the first postoperative months, the expected (hydrogen) gas formation can be observed within the bone and the soft tissues around the implant due to the corrosion process of the Mg implant. In long-term follow-up, the gas formation disappears, and the Mg screw can be visualized as a decreasing silhouette. In MRI, the screw appeared as a thin linear hypo-intensity in the third year after implantation in a clinical study with hallux valgus corrections [16]. However, the experience in assessing Mg imaging properties is limited due to the innovativeness of the implant material. In particular, little is known about imaging characteristics of Mg screws in the course of time after implantation. Thus, this study aimed to investigate changes of MRI artifacts as an indication of the degradation process of Mg screws.

## Materials and methods

### Patients and study design

A retrospective review study was performed on all patients with either MM fracture or MM osteotomy who underwent surgical fixation with bioabsorbable Mg screws between February 2015 and June 2019 and who had postoperative MRI examinations at various time intervals during follow-up in the authors’ institution. The institutional review board approved the study protocol (IRB approval number: 2019-139/12.01). The study was carried out in accordance with the ethical standards laid down in the 1964 Declaration of Helsinki and its later amendments. Seven female and 10 male patients with a mean age of 33.6 ± 13.9 years (min-max, 17–56 years) were included. The diagnosis was a MM fracture in five cases, and the rest of the 12 patients underwent MM osteotomy for an OLT. Among the ankle fractures, four had isolated MM fracture, and one patient had a bimalleolar fracture. Six patients underwent a second postoperative MRI, two of them had a third MRI. Detailed patient characteristics are presented in Table 1. The reason for MRI examination was ankle pain and monitoring the healing process of the mosaicplasty procedure, and imaging of the cartilage. None of the patients had signs of infection or osteomyelitis.

**Table 1:** Demographic and clinical characteristics of patients.

Case #	Age, years	Sex	Side	Diagnosis	1st MRI	2nd MRI	3rd MRI
					Months after operation		
1	21	M	L	Isolated MM fracture	30^a^		
2	20	F	R	Bimalleolar fracture	43^a^		
3	24	M	L	Isolated MM fracture	19^b^		
4	20	M	L	Isolated MM fracture	11^b^		
5	45	M	L	Isolated MM fracture	2^a^		
6	51	F	L	OLT	12^a^		
7	53	F	L	OLT	3^a^	13^b^	
8	23	M	L	OLT	3^a^	14^a^	36^b^
9	47	M	L	OLT	48^a^		
10	56	M	L	OLT	48^a^		
11	51	F	R	OLT	2^a^	13^b^	
12	36	F	R	OLT	6^a^	10^b^	
13	24	M	R	OLT	1^a^	11^a^	
14	17	F	R	OLT	5^a^	13^a^	30^b^
15	35	M	R	OLT	32^a^		
16	30	M	L	OLT	12^b^		
17	19	F	L	OLT	9^b^		

M, male; F, female; R, right; L, left; MM, medial malleolar; OLT, osteochondral lesion of the talus. ^a^1.5T MRI. ^b^3T MRI.

### Surgical procedures and implants

**Figure 1:**
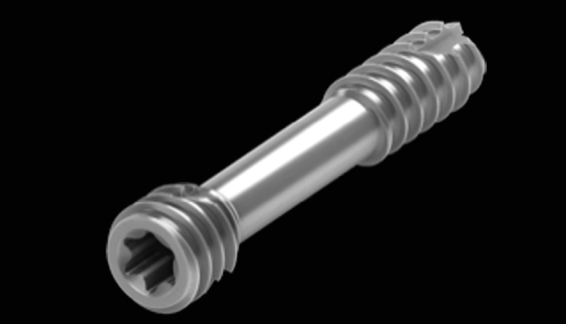
Image of the MAGNEZIX^®^ compression screw with a 3.2 mm diameter.

All patients were operated under spinal anesthesia and tourniquet control in a supine position. A medial longitudinal incision was used for the surgical approach. In both MM fracture and osteotomy cases, first, the anatomic reduction was achieved, and two parallel guide wires perpendicular to the fracture/osteotomy line were inserted for temporary fixation. The fracture was fixed with two bioabsorbable Mg headless compression screws. The screws were countersunk to the cortical bone surface until sufficient compression was obtained. Bioabsorbable Mg screws (MAGNEZIX^®^ CS, Syntellix AG, Hanover, Germany), which are made of the Mg alloy MgYREZr, were used for the fixation (Figure 1). These screws were designed similar to Herbert screws to provide compression between the fracture fragments. The diameter of all screws was 3.2 mm, the screws’ length was chosen in accordance with the pattern of fracture or the size of the fragment and the surgeon’s preference, ranging 28–40 mm.

### Image acquisition and post-processing

Patients were referred to MRI imaging at different time points according to clinical presentation. MRI scans were performed on two MRI units: 1.5T MRI Achieva DS Advance (Philips Healthcare) and 3T MRI Ingenia (Philips Healthcare) using a 16-channel head or an 8-channel ankle coil respectively. Sequences followed a standard ankle protocol from clinical routine comprising proton density weighted (PDw) spectral presaturation inversion recovery (SPIR) sequences, a T1w turbo spin echo (TSE) sequence, and a T2w SPIR sequence (for detailed scan parameters: Table 2). All images were transferred to a client server-based picture archiving and communication system (PACS) digital workstation (Sectra IDS7, Ver. 18.2., Sectra AB, Sweden).

**Table 2:** MRI acquisition parameters.

MRI parameters		T2w SPIR	T1w TSE	PDw SPIR	PDw SPIR
Orientation		Sagittal	Sagittal	Axial	Coronal
Repetition time, ms	1.5T	3,557	450	4,500	3,881
	3.0T	3,038	498	2,926	2,768
Echo time, ms	1.5T	50	20	30	30
	3.0T	80	12	30	30
Receiver bandwidth, hertz/pixel	1.5T	221.2/0.982	347.9/0.624	228.6/0.950	212.9/1.020
	3.0T	129.2/3,362	241.8/1,796	195.1/2,226	179.8
Flip angle, °	1.5T	90	90	90	90
	3.0T	90	90	90	90
Field-of-view, mm^2^	1.5T	130	130	128	130
	3.0T	150	150	150	160
Matrix	1.5T	200 × 160	268 × 210	216 × 173	228 × 175
	3.0T	236 × 189	292 × 255	228 × 143	256 × 159
Slice thickness/gap, mm	1.5T	0.4/0	3.5/0.35	0.4/0	0.4/0
	3.0T	3/0.3	3/0.3	3/0.3	3/0.3
Pixel dimensions, mm^2^	1.5T	0.56	0.31	0.39	0.40
	3.0T	0.36	0.20	0.31	0.31
Number of excitations	1.5T	2	2	2	2
	3.0T	2	2	2	2
Number of slices	1.5T	16	16	26	26
	3.0T	25	25	25	30
Phase encoding direction	1.5T	COL	COL	COL	COL
	3.0T	ROW	ROW	ROW	ROW
Phase sampling, %	1.5T	80	78.4	80.2	77
	3.0T	80	87.4	78.4	75.2

SPIR, spectral presaturation inversion recovery; TSE, turbo spin echo; PDw, proton density weighted; COL, columns; ROW, rows; MRI, magnetic resonance imaging.

### MRI assessment and measurements

**Figure 2:**
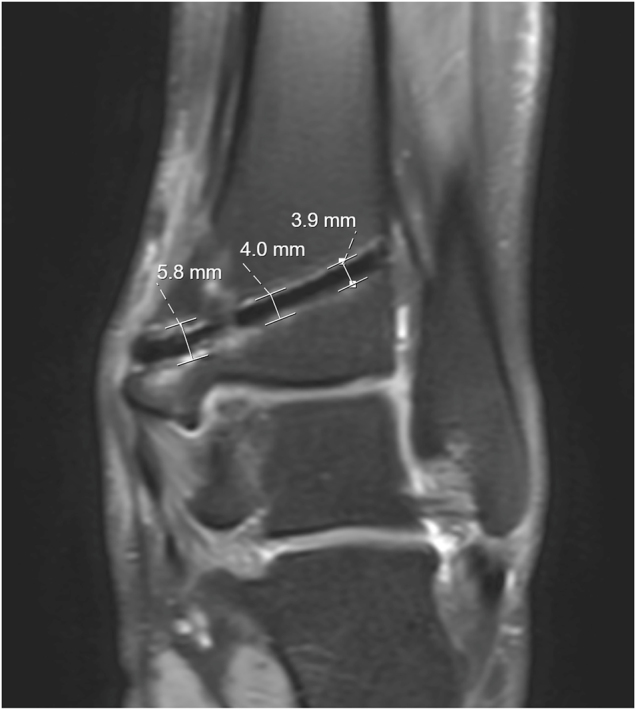
Measurement of artifact extent around the screw on a proton density weighted (PDw) coronal MR image.

Images were assessed quantitatively by measuring the area of signal loss at three predefined locations (proximal, middle, distal) of both implanted screws. The size of the signal loss artifact was measured and recorded in millimeters (mm) on the T1w sagittal and PDw coronal planes (Figure 2). In addition, two independent radiologists with 4 and 5 years of experience semi-quantitatively ranked other artifacts (such as signal distortions or pile-up artifacts) generated by the screws using a five-point Likert scale (1 = no artifacts, 2 = minimal artifacts, 3 = considerable artifacts, 4 = artifacts impairing diagnostic validity, 5 = no interpretation of surrounding tissue possible). In case of follow-up MRI, readers were encouraged to compare the artifacts in one patient over time. An additional qualitative evaluation included the assessment of common postoperative changes, such as bone marrow edema (BME), changes in the surrounding tissue or depictability of the directly adjacent anatomical structures.

### Statistical analysis

Quantitative variables were reported as mean ± standard deviation (SD). Interreader agreement for the measurements was calculated using Cohen’s kappa. For the estimation of artifact decrease, a robust linear mixed-effects model (RLME) was applied. To compare the semi-quantitative Likert scales, a cluster bootstrap analysis was performed. The type I error level was set to 0.01. Analyses were performed using R software (version 4.0.29).

## Results

Mg screws were visible in all MRI sequences and could be depicted as a signal-loss artifact within the surrounding bone. The readers showed an almost perfect agreement: κ=0.88 (p<0.001). In the first postoperative MRIs, the mean size of the signal loss area was measured 5.4 ± 3.2 mm for PDw coronal and 5.2 ± 2.1 mm for T1w sagittal sequences. In six patients, the artifact was measured in a second postoperative MRI 4.9 ± 2.0 mm for PDw coronal and 4.2 ± 1.0 mm for T1w sagittal sequences. A third postoperative MRI was obtained in two patients, and the artifact was measured 2.7 ± 0.8 mm in PDw coronal and 2.9 ± 0.7 mm in T1w sagittal sequences. Table 3 gives detailed measurement results depending on MR field strength and sequence.

**Table 3:** Results of artifact measurements of all postoperataive MRIs for proton density weighted (PDw) coronal and T1 weighted sagittal sequences for 1.5 T and 3 T MRI respectively.

		1st MRI	2nd MRI	3rd MRI
Numbers of MRI (1.5T/3 T)		(13/4)	(3/3)	(0/2)
Mean time interval between operation and MRI in months		16.8 (1–48)	12.3 (10–14)	33 (30–36)
1.5T MRI	PDw coronal	5.9 ± 3.5	5.8 ± 2.1	–
	T1w sagittal	5.3 ± 2.4	4.2 ± 0.8	–
3T MRI	PDw coronal	3.9 ± 0.7	4.0 ± 1.4	2.7 ± 0.8
	T1w sagittal	4.8 ± 0.9	4.3 ± 1.1	2.9 ± 0.7
1.5T + 3T MRI	PDw coronal	5.4 ± 3.2	4.9 ± 2.0	2.7 ± 0.8
	T1w sagittal	5.2 ± 2.1	4.2 ± 1.0	2.9 ± 0.7

PDw, proton density weighted; T1w, T1 weighted; MRI, magnetic resonance imaging.

**Figure 3:**
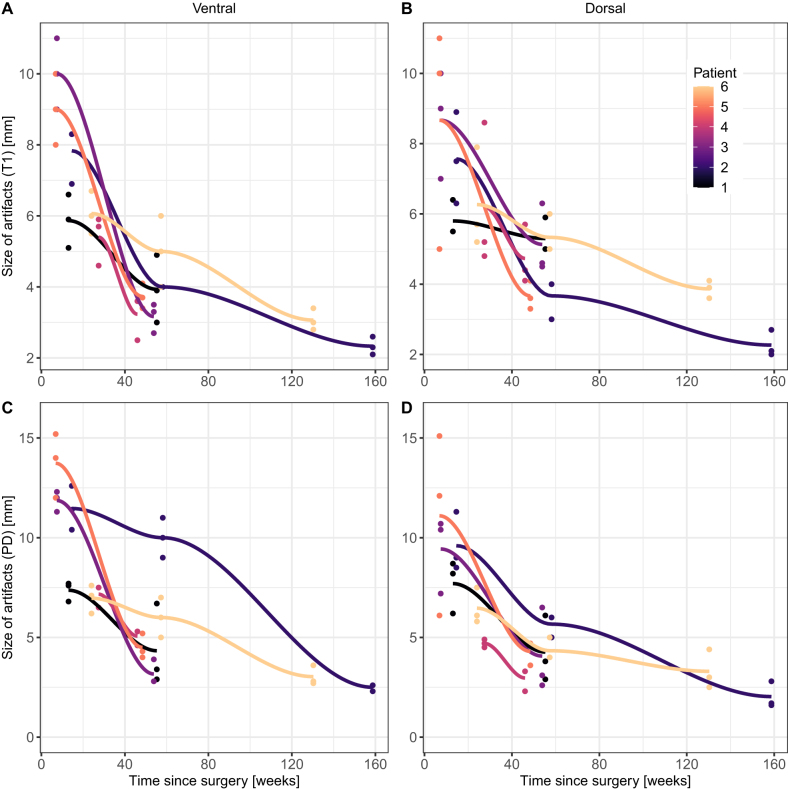
Artifact extent of the ventral (A and C) and dorsal (B and D) Mg screw in six patients at different time points after surgery. The extent of the signal loss area of the Mg screw (y-axis) decreases both in T1 weighted (A and B) and proton density weighted (PDw) sequences (C and D) over the time (x-axis).

**Figure 4:**
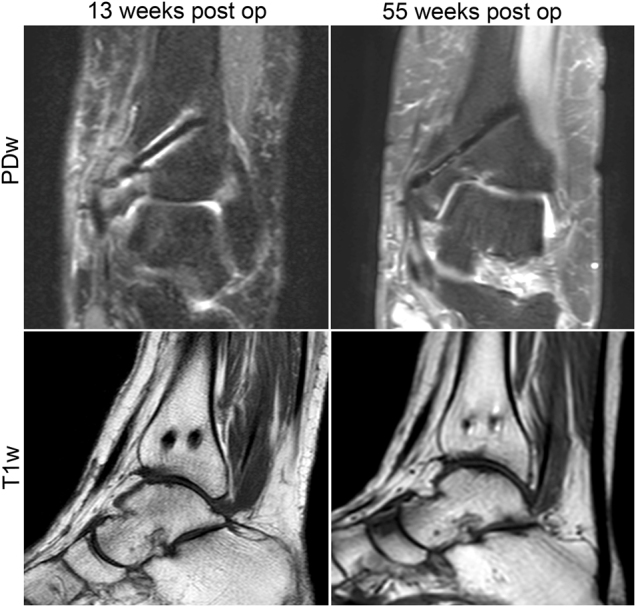
Artifact extent in one patient at two different time points is shown for proton density (PDw) and T1 weight sequences. After 55 weeks (right side) the extent of the artifact is reduced compared to 13 weeks postoperatively (left side).

Figure 3 shows selected measurement results of the patients with two (n=6) and three (n=2) follow-up MRIs at different time points given in weeks for the dorsal and ventral screws in PDw and T1w images, respectively. In these patients the artifact decreased significantly by 0.058 mm (95% confidence interval [CI] 0.049–0.072; p<0.001) in PDw and by 0.036 mm (95% CI 0.030–0.047; p<0.001) in T1w per week. Figure 4 illustrates the decrease of artifact extent in one patient 13 weeks and 55 weeks postoperatively.

**Figure 5:**
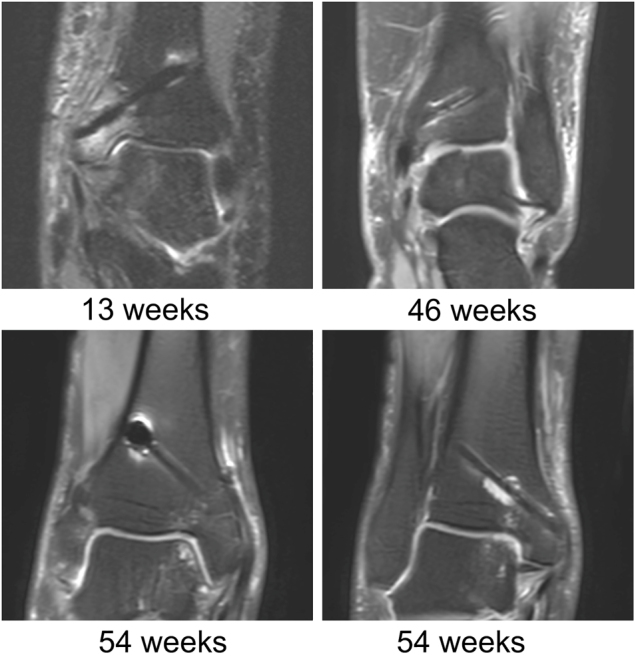
Examples of MRI findings in proton density weighted (PDw) sequences for different time points and patients: bone marrow edema in the medial malleolus (13 weeks postoperatively), visibility of a hyperintense line in the screw center (46 weeks postoperatively), gas accumulation at the screw tip (54 weeks postoperatively), and liquid collection around the screw (54 weeks postoperatively).

Both readers rated artifact strength on the five-point Likert scale to be lower at follow-up. In detail, the mean artifact rating decreased by 1.78 points (95% CI: 1.05–2.36; p=0.008) from the first postoperative MRI to the follow-up controls. Both readers reported different findings without any certain time dependency on the postoperative time interval. Table 4 gives an overview of the reported findings. Figure 5 visualizes some important findings in different patients.

**Table 4:** Overview of the reported findings with time intervals after surgery.

Reported finding	Reported time intervals in different patients
Bone marrow edema (medial malleolus)	2 months
	3 months (disappearance in month 13)
	10 months
	14 months
Bone marrow edema (talus)	3 months (decreasing in month 14, increasing again in month 36)
	13 months
Edema of the surrounding soft tissue	3 months (decreasing in month 13)
Gas collection	13 months
	1 month (disappearance in month 13)
Liquid collection	13 months
	13 months (disappearance in month 30)
Hyperintense T2-line in screw center	36 months

## Discussion

In this retrospective study, longitudinal postoperative MRIs of patients who had a biodegradable Mg screw implanted for MM fixation were analyzed. The artifact size in the early period MRI images was larger than in the late period MRIs. In follow-up MRIs within the same patient, measurements showed decreased artifact size. Some MRIs also showed gas formation, surrounding edema, or liquid formations. These findings did not correlate with clinical symptoms and were mostly decreasing over the time.

MRI artifacts near metallic implants arise from local magnetic field inhomogeneities due to considerable differences between the magnetic properties of the human tissue and those of the implanted metal. This leads to large resonant frequency changes and failure of many MRI mechanisms. The appearance of typical metal artifacts in MRI includes signal loss, failure of fat suppression, geometric distortion, blooming, and bright pile-up artifacts [21], [22], [23]. The lower mass magnetic susceptibility of Mg (6.9 × 10^−9^ m^3^/kg) compared to titanium (4.01 × 10^−8^ m^3^/kg) [24] explains why the screw borders appeared sharply demarcated and the artifact was mainly limited to a signal-loss area with a thin bright pile-up margin without significant geometric distortions or blooming artifacts in all postoperative controls in the present study. These findings correspond to *ex vivo* studies that reported Mg screws generating significantly fewer artifacts in MRI compared to conventional titanium [19, 20].

In this study, we measured a decrease of artifact size within the same patient over time, especially the signal-loss area became thinner the more weeks passed since implantation. A likely explanation for these findings is that the amount of pure Mg metal in the screw decreases over time because Mg is an active metal and the implant starts to corrode with the contact of body fluids, particularly with water. The longer the MRI examination was performed after implantation, the fewer artifacts were observed due to a constant decrease of metal content within the screw. Our observations indicate that the implant is bioactive and that it has been replaced by a material with weaker magnetic properties. This assumption is supported by the fact, that in our study a total of five follow-up MRIs was performed at a 3T MR unit, while the compared first postoperative scans were carried out at a 1.5T MRI. Even though metal artifacts are expected to be greater at stronger field strengths, the measurements of artifact extent decreased over the time and did not seem to be influenced by the scan conditions.

Our results are in concordance with previous radiographic findings in clinical studies with Mg screws used for chevron osteotomy fixation in hallux valgus patients [13], [14], [15], [16], MM osteotomy [17] or fracture fixation [18]. Over the time, the screws showed a reduced attenuation of X-rays, which is in line with the degradation process seen in Mg screws. On radiographs that were taken two years postoperatively, the Mg screw was described as almost unidentifiable [13].

Some MRIs in our study showed a T2-hyperintense signal in the central screw canal or directly adjacent to the outer screw border, consistent with liquid collections. A signal loss area remained in the position of the screw and most likely corresponds to residual Mg as well as newly formed peri-implant bone, as suggested in histological analysis in animal studies [25]. Previous histological analysis in a rabbit model could demonstrate the degradation process, and moreover, newly formed bone trabeculae were observed to be grown into parts of the degraded material. The results of energy-dispersive X-ray (EDX) analysis suggested the remaining implant (MgYREZ screw) consisting mainly of substances similar to an apatite formation, and µCT scans also indicated a material change [26]. Besides bone formation at the implant surface, a recent animal study [27] reported the formation of some lacunae close to the Mg implants, maybe equivalent to the liquid collections in our study.

Clinical studies [13], [14], [15], [16], [17], [18] reported radiographic radiolucency around the Mg implants, which occurred a few weeks after the operation, but completely disappeared on follow-up X-ray. These radiolucencies could not be reproduced in our MRI study, although the time intervals were comparable. In general, based on imaging in clinical studies, it remains unclear what exactly happens at the bone-implant interface and how much of the Mg is replaced by bone tissue in humans. It is essential to fully understand the corrosion process.

The body fluids around the Mg implant contain chlorine, calcium, and phosphate ions, which trigger the production of different Mg_x_Ca_y_(PO4)z(OH)n compounds such as calcium phosphate and/or calcium Mg phosphate [28]. These are considered complex bioactive mineral products that form a deposition layer on the Mg implant surface, and their exact influence on imaging artifacts is unknown. Moreover, there is a release of hydroxide ions from Mg hydroxide upon the reaction with chlorine ions resulting in a local increase in pH value around the implant during the corrosion process [29, 30]. Since the recorded MRI signal is predominantly based on protons, a distinct increase in pH value could influence the signal, which would not happen with conventional implants.

Furthermore, in some cases, we observed a BME around the screw. From a radiological perspective, a BME is defined as an altered signal area, which shows an intermediate or low signal intensity on T1w and a high signal intensity on T2w compared to normal bone marrow [31]. While the MRI appearance of BME itself is relatively consistent, the associated pathologies may vary (e.g., trauma-induced, inflammatory, ischemic) [31, 32]. In our study, the BME was observed a few weeks after the operation and disappeared later on. For a more specific chronological association, additional prospective studies with a greater number of cases are needed. Analogously to the radiolucent line, the BME showed no apparent association with clinical symptoms or infection signs. It can be assumed that BME is an unspecific reaction towards the degradation process of the Mg implant. It remains a challenge to identify the exact local cellular processes triggered by the Mg corrosion products, such as local changes in the capillary wall, intravascular pressure changes, influence on lymphatic drainage, or osmotic changes due to a local increase of Mg ions. Although histological evaluations in rabbits after anterior cruciate ligament reconstruction revealed neither inflammatory reactions nor necrosis of the tendon in the course of the normal degradation process [33], it is very difficult to perform a direct correlation of a BME finding on MRI and a histopathologic examination, especially in a clinical study. The correct interpretation of the BME around Mg implants thus remains challenging. The residual metallic Mg material makes it difficult to apply additional sequences like diffusion-weighted imaging (DWI) or chemical shift-weighted imaging (CSWI) as they are prone to metal artifacts.

Another relevant aspect concerning Mg implants is gas formation. The degradation process produces hydrogen gas, and gas cavities have been reported in previous studies [14], [15], [16]. Gas evolution has been described starting immediately after implantation of Mg screws, and during the first two months, variable amounts of gas were observed in the surrounding soft tissue [13, 17]. In the course of time, the gas was absorbed and was no longer visible later than the third month on plain radiographs [13]. Hence, it is important not to misinterpret this finding as a gas-producing infection and mislead the surgeon to unnecessary revision surgery. µCT scans in an animal study showed gas liberation being the most prominent four weeks after implantation and significantly decreasing by 24 weeks [33]. In another study with intraarticularly implanted Mg pins in rabbits, radiographs showed the accumulation of gas most pronounced after four weeks, which decreased after 12 weeks with the explanation of diffusion processes and transportation via the vascular system [34]. In this study, we observed several gas accumulations at different time points adjacent to the Mg screw. On additional follow-up MRIs, these gas formations were no longer detectable.

One limitation of this study is its relatively small group of subjects with a few numbers of follow-up MRIs. Due to the retrospective study nature of this work, several factors that may change the artifact size and shape could not be controlled, including the imaging protocol, MRI device, patient position, and the examination timing and intervals. This might be important as artifact shape varies substantially depending on the MRI’s field strength or on the exact screw position within the scanner regarding the scanner axis. Furthermore, individual healing capabilities and responses towards the degradation process could not be assessed.

In summary, Mg implants create fewer artifacts than other metallic implants, which facilitates the assessment of the surrounding tissue, especially the bone-implant interface. The size of the artifact is reduced over time, which might be an indirect finding supporting the degradation of Mg implants. However, Mg implants provide unusual imaging characteristics and must not be evaluated employing conventional rules for other metallic implants in orthopedic surgery. The degradation process needs to be fully understood so that findings, such as edema or gas formation, which naturally occur during the degradation process of the screw, are not misinterpreted. More experience is needed to better understand the appearance of a premature implant loosening or implant failure of Mg implants in MRI. Other techniques might be helpful in this direction. For example, MR spectroscopy can analyze the chemical composition of tissues and could detect small metabolite concentrations *in vivo*. Novel MRI methods that are based on pH-sensitive T1 relaxivity and are used for pH value measurement, e.g., within tumor masses, may be used to investigate pH changes [35]. These research questions need to be answered with well-designed studies in the future.

## Supplementary Material

Supplementary Material

## References

[1] Kannus P, Palvanen M, Niemi S, Parkkari J, Järvinen M (2002). Increasing number and incidence of low-trauma ankle fractures in elderly people: Finnish statistics during 1970-2000 and projections for the future. Bone.

[2] Ebraheim NA, Ludwig T, Weston JT, Carroll T, Liu J (2014). Comparison of surgical techniques of 111 medial malleolar fractures classified by fracture geometry. Foot Ankle Int.

[3] Barg A, Pagenstert G, Leumann A, Valderrabano V (2013). Malleolar osteotomy--osteotomy as approach. Orthopä.

[4] Muir D, Saltzman CL, Tochigi Y, Amendola N (2006). Talar dome access for osteochondral lesions. Am J Sports Med.

[5] Barcak EA, Beebe MJ, Weinlein JC (2018). The role of implant removal in orthopedic trauma. Orthop Clin N Am.

[6] Fenelon C, Murphy EP, Galbraith JG, Kearns SR (2019). The burden of hardware removal in ankle fractures: how common is it, why do we do it and what is the cost? A ten-year review. Foot Ankle Surg.

[7] Naumann MG, Sigurdsen U, Utvåg SE, Stavem K (2016). Incidence and risk factors for removal of an internal fixation following surgery for ankle fracture: a retrospective cohort study of 997 patients. Injury.

[8] Leumann A, Horisberger M, Buettner O, Mueller-Gerbl M, Valderrabano V (2016). Medial malleolar osteotomy for the treatment of talar osteochondral lesions: anatomical and morbidity considerations. Knee Surg Sports Traumatol Arthrosc Off J ESSKA.

[9] Li Z-H, Yu A-X, Guo X-P, Qi B-W, Zhou M, Wang W-Y (2013). Absorbable implants versus metal implants for the treatment of ankle fractures: a meta-analysis. Exp Ther Med.

[10] Böstman OM, Pihlajamäki HK (2000). Adverse tissue reactions to bioabsorbable fixation devices. Clin Orthop.

[11] Mosier-Laclair S, Pike H, Pomeroy G (2001). Intraosseous bioabsorbable poly-L-lactic acid screw presenting as a late foreign-body reaction: a case report. Foot Ankle Int.

[12] Seitz J-M, Lucas A, Kirschner M (2016). Magnesium-based compression screws: a novelty in the clinical use of implants. JOM.

[13] Acar B, Kose O, Turan A, Unal M, Kati YA, Guler F (2018). Comparison of bioabsorbable magnesium versus titanium screw fixation for modified distal chevron osteotomy in hallux valgus. BioMed Res Int.

[14] Plaass C, von Falck C, Ettinger S, Sonnow L, Calderone F, Weizbauer A (2018). Bioabsorbable magnesium versus standard titanium compression screws for fixation of distal metatarsal osteotomies - 3 year results of a randomized clinical trial. J Orthop Sci Off J Jpn Orthop Assoc.

[15] Windhagen H, Radtke K, Weizbauer A, Diekmann J, Noll Y, Kreimeyer U (2013). Biodegradable magnesium-based screw clinically equivalent to titanium screw in hallux valgus surgery: short term results of the first prospective, randomized, controlled clinical pilot study. Biomed Eng Online.

[16] Plaass C, Ettinger S, Sonnow L, Koenneker S, Noll Y, Weizbauer A (2016). Early results using a biodegradable magnesium screw for modified chevron osteotomies. J Orthop Res.

[17] Acar B, Kose O, Unal M, Turan A, Kati YA, Guler F (2020). Comparison of magnesium versus titanium screw fixation for biplane chevron medial malleolar osteotomy in the treatment of osteochondral lesions of the talus. Eur J Orthop Surg Traumatol Orthop Traumatol.

[18] May H, Alper Kati Y, Gumussuyu G, Yunus Emre T, Unal M, Kose O (2020). Bioabsorbable magnesium screw versus conventional titanium screw fixation for medial malleolar fractures. J Orthop Traumatol Off J Ital Soc Orthop Traumatol.

[19] Sonnow L, Könneker S, Vogt PM, Wacker F, von Falck C (2017). Biodegradable magnesium Herbert screw - image quality and artifacts with radiography, CT and MRI. BMC Med Imag.

[20] Filli L, Luechinger R, Frauenfelder T, Beck S, Guggenberger R, Farshad-Amacker N (2015). Metal-induced artifacts in computed tomography and magnetic resonance imaging: comparison of a biodegradable magnesium alloy versus titanium and stainless steel controls. Skeletal Radiol.

[21] Farrelly C, Davarpanah A, Brennan SA, Brennan S, Sampson M, Eustace SJ (2010). Imaging of soft tissues adjacent to orthopedic hardware: comparison of 3-T and 1.5-T MRI. AJR Am J Roentgenol.

[22] Hargreaves M (2000). Skeletal muscle metabolism during exercise in humans: exercise metabolism. Clin Exp Pharmacol Physiol.

[23] Radzi S, Cowin G, Robinson M, Pratap J, Volp A, Schuetz MA (2014). Metal artifacts from titanium and steel screws in CT, 1.5T and 3T MR images of the tibial Pilon: a quantitative assessment in 3D. Quant Imag Med Surg.

[24] Kammer C (2000). Magnesium-Taschenbuch.

[25] Lindtner RA, Castellani C, Tangl S, Zanoni G, Hausbrandt P, Tschegg EK (2013). Comparative biomechanical and radiological characterization of osseointegration of a biodegradable magnesium alloy pin and a copolymeric control for osteosynthesis. J Mech Behav Biomed Mater.

[26] Waizy H, Diekmann J, Weizbauer A, Reifrath J, Bartsch I, Neubert V (2014). In vivo study of a biodegradable orthopedic screw (MgYREZr-alloy) in a rabbit model for up to 12 months. J Biomater Appl.

[27] Naujokat H, Ruff CB, Klüter T, Seitz J-M, Açil Y, Wiltfang J (2020). Influence of surface modifications on the degradation of standard-sized magnesium plates and healing of mandibular osteotomies in miniature pigs. Int J Oral Maxillofac Surg.

[28] Jang Y, Collins B, Sankar J, Yun Y (2013). Effect of biologically relevant ions on the corrosion products formed on alloy AZ31B: an improved understanding of magnesium corrosion. Acta Biomater.

[29] Gu X, Zhou W, Zheng Y, Dong L, Xi Y, Chai D (2010). Microstructure, mechanical property, bio-corrosion and cytotoxicity evaluations of Mg/HA composites. Mater Sci Eng C.

[30] Wong HM, Yeung KWK, Lam KO, Tam V, Chu PK, Luk KDK (2010). A biodegradable polymer-based coating to control the performance of magnesium alloy orthopaedic implants. Biomaterials.

[31] Starr AM, Wessely MA, Albastaki U, Pierre-Jerome C, Kettner NW (2008). Bone marrow edema: pathophysiology, differential diagnosis, and imaging. Acta Radiol (Stockh) 1987.

[32] Hofmann S, Kramer J, Vakil-Adli A, Aigner N, Breitenseher M (2004). Painful bone marrow edema of the knee: differential diagnosis and therapeutic concepts. Orthop Clin N Am.

[33] Diekmann J, Bauer S, Weizbauer A, Willbold E, Windhagen H, Helmecke P (2016). Examination of a biodegradable magnesium screw for the reconstruction of the anterior cruciate ligament: a pilot in vivo study in rabbits. Mater Sci Eng C Mater Biol Appl.

[34] Ezechieli M, Diekmann J, Weizbauer A, Becher C, Willbold E, Helmecke P (2014). Biodegradation of a magnesium alloy implant in the intercondylar femoral notch showed an appropriate response to the synovial membrane in a rabbit model *in vivo*. J Biomater Appl.

[35] Garcia‐Martin ML, Martinez GV, Raghunand N, Sherry AD, Zhang S, Gillies RJ (2006). High resolution pHe imaging of rat glioma using pH-dependent relaxivity. Magn Reson Med.

