# Supplementary material for: Alterations in magnetic resonance imaging characteristics of bioabsorbable magnesium screws over time in humans: a retrospective single center study
Source: Innov Surg Sci. 2021 Dec 23;6(3):105–13. doi: 10.1515/iss-2021-0032 (PMC8826160; doi:10.1515/iss-2021-0032)
Supplement: Supplementary file 1 — Supplementary Material [file j_iss-2021-0032_suppl.pdf]

Reviewer Assessment

L. Sonnow et al.: Alterations in magnetic resonance imaging characteristics of bioabsorbable magnesium screws over time in humans: a retrospective single center study

Reviewers' Comments to Original Submission

Reviewer 1: annonymous

Date received: 7-Sep-2021
Reviewer recommendation: **Return to author for major modifications**
Reviewer overall scoring: **Low**

Assessment Form scores: 5 = High/Yes; 3 = Medium/Adequate; 1 = Low

Is the subject area appropriate for the journal		4			
Does the title clearly reflect the paper's content?		4			
Does the abstract clearly reflect the paper's content			3		
Do the keywords clearly reflect the paper's content?	5				
Does the introduction present the problem clearly?		4			
Are the results/ conclusions justified?					1
How comprehensive and up-to-date is the subject matter presented?	5				
How adequate is the data presentation?				2	
Are units and terminology used correctly?		4			
Is the number of cases adequate?			3		
Are the experimental methods/ clinical studies adequate?		4			
Is the length appropriate in relation to the content?		4			
Does the reader get new insights from the article?		4			
Please rate the practical significance.		4			
Please rate the accuracy of methods.					1
Please rate the statistical evaluation and quality control.		4			
Please rate the appropriateness of the figures and tables.		4			
Please rate the appropriateness of the references.		4			
Please evaluate the writing style and use of language.				2	
Please judge the overall scientific quality of the manuscript.				2	
Are the methods used worthy of reproduction in greater deal?	Yes				
Would you be willing to review a revision of this manuscript?	Yes				

Comments to author: This study describes postoperative MRI changes after insertion of bioabsorbable malleolar screw. This is quite of interest not only histologically described in previous studies but also in MRI imaging, as these postoperative changes has to differentiated from signs of implant failure and loosening. However, the study has some major issues which has to be addressed. Quantitative analysis from 3T and 1.5T MRI are mixed together inter-individually and intra-individually. It should be clear that one of the factors which influences the artifact extent most is the magnetic field strength. Therefore, a re-evaluation of the results is indispensable. The signal loss/changes in MRI are declared as artifacts, but this is not applicable. The signal loss comprises the screw itself, blooming artifacts, pile up artifacts. The precise formulation is either to describe signal loss/changes or make a dedicated evaluation what is the real extent of artifact and what is screw itself by e.g., measuring the screws on X-ray. The result section is quite missing results. Some results are mixed up in the discussion section.
Moreover, a profound language editing is necessary. Wrong numbering of tables in text.

Abstract:
Methods not cleary described. Though 17 patients were included into the study only six patients received a second MRI. The data of the six patients and not 17 are the main results in this study and might be misleading.
As mentioned above mixing values from measurements or signal changes from 3T and 1.5 T MRI inter-individually and intra-individually does not show valid results.
P1 line 28: wrong order of units. Correct: 0.04 mm (CI: 0.03-0.05).
P1 line 31: wording: not artifact strength, but artifact extent
P1 line 35: Cohen's kappa>0.9. Please give exact value.
Key words: wording: not metallic artifacts, but metal artifact
Abbreviations: Capitalization of the word "X-ray" please!

Main Document
P5 line 16: bone instead of bony
P5 line 22: add "and thus, implant-related…."
P5 line 39: Split it up in two sentences: ….implants. However,
P5 line 45: Please use no space or non-breaking space between end of sentence and citation. Then, there would not be a line break between those.
P5 line 54: "unique" might be exaggerated.
P5 line 56: Wording: it is not "lesser" density. To be correct: "reduced attenuation of X-rays"
P6 line 20: Syntax: Thus, this study
P6 line 33: what are the exclusion criteria. Why were 31 patients out of 48 patients excluded?
P6 line 58: wrong numbering of tables.
P7 line 9: Wording: It is not a 1.5 Tesla. It is 1.5 T MRI or Magnetic field strength.
Table 1: What is the time interval between operation and 1st MRI, 2nd MRI, 3rd MRI?

P8 line 33: wording: better or something similar: Patients were referred to MRI imaging at different time points according clinical presentation.
P8 line 39: non-breakig space between 3.0 and T MRI Ingenia.
P9 line 59: Abbreviation COL not described
P10 line 3: Abbreviation ROW not described
P10 line 14: As described above it is not measurements of artifact, but area of signal loss.
P10 line 18: perpendicular measurements only applicable on images in coronar and sagittal orientation. Perpendicular measurements to the screw long axis on axial images not possible as the axial images are oriented to the ankle joint not axial to the screw.
P10 line 25: years of experience?

Results:
Missing information of qualitative results. How many patients had bone marrow edema, soft tissue changes etc. What is the mean time interval between operation and imaging? This is quite important to evaluate postoperative changes.
P10 line 58: non-breaking space between 2.1 and mm.

Invalid quantitative results as comparing measurements from 1.5T and 3.0 T MRI images.

Discussion:
Extensive and good discussion of the topic.
P12 Line 20: What is the clinical impact.
P12 line 54: particularly “with” water
P12 line 56: switch of tempus.
P14 line 20: when exactly did the qualitative changes disappeared. It is not described in the result section.

Table legends:
Where are table 1-3 if the tables start with table 4?
P20 line 18: wording: not scanning parameters, but acquisition parameters

Figure legends
P21 line 8: wording: not “appearance” but maybe: Image of a MAGNEZIX R compression with 3.2 mm diameter
P21 line 18: not artifact sizes! Artifact extent.
P21 line 31: wording: different MRI..different time points...different patients.

Figures:
Figure 3: Please more extensive description.
Figure 4: What does the images show. Please describe e.g., “Reduced extent of artifacts in image a in comparison to image b”....
Figure 5: what is seen on these beautiful images. Please describe findings in the figure caption

Reviewer 2: annonymous

Date received: 09-Sep-2021
Reviewer recommendation: **Return to author for minor modifications**
Reviewer overall scoring: **High**

Assessment Form scores: 5 = High/Yes; 3 = Medium/Adequate; 1 = Low

Is the subject area appropriate for the journal		4			
Does the title clearly reflect the paper's content?	5				
Does the abstract clearly reflect the paper's content	5				
Do the keywords clearly reflect the paper's content?	5				
Does the introduction present the problem clearly?		4			
Are the results/ conclusions justified?		4			
How comprehensive and up-to-date is the subject matter presented?	5				
How adequate is the data presentation?	5				
Are units and terminology used correctly?	5				
Is the number of cases adequate?			3		
Are the experimental methods/ clinical studies adequate?		4			
Is the length appropriate in relation to the content?			3		
Does the reader get new insights from the article?	5				
Please rate the practical significance.		4			

Please rate the accuracy of methods.		4			
Please rate the statistical evaluation and quality control.	5				
Please rate the appropriateness of the figures and tables.	5				
Please rate the appropriateness of the references.	5				
Please evaluate the writing style and use of language.		4			
Please judge the overall scientific quality of the manuscript.		4			
Are the methods used worthy of reproduction in greater deal?	Yes				
Would you be willing to review a revision of this manuscript?	Yes				

Comments to author: Thank you very much for this interesting work. The questioning, argumentation and presentation of your results are well done.
Introduction: Can you clarify, whether there is a recommendation (guideline) for implant removal in adults? For children, there is such a guideline (DGOUC) recommending the implant removal.
In Table 1: Can you insert the reason for the MRI for each case?. You have to clarify, that the patient was bone healthy and had no signs of osteomyelitis …

Authors' Response to Reviewer Comments

Date received: 20-Sep-2021

Response to reviewer 1

Dear Reviewer 1, thank you for the time and effort spent on reviewing our manuscript. All of your remarks were considered and included into the revised version. All changes are highlighted in yellow. In addition, a profound language editing was performed.
Point-by-point revision:

This study describes postoperative MRI changes after insertion of bioabsorbable malleolar screw. This is quite of interest not only histologically described in previous studies but also in MRI imaging, as these postoperative changes has to differentiated from signs of implant failure and loosening. However, the study has some major issues which has to be addressed. Quantitative analysis from 3T and 1.5T MRI are mixed together inter-individually and intra-individually. It should be clear that one of the factors which influences the artifact extent most is the magnetic field strength. Therefore, a re-evaluation of the results is indispensable.The signal loss/changes in MRI are declared as artifacts, but this is not applicable. The signal loss comprises the screw itself, blooming artifacts, pile up artifacts. The precise formulation is either to describe signal loss/changes or make a dedicated evaluation what is the real extent of artifact and what is screw itself by e.g., measuring the screws on X-ray. The result section is quite missing results. Some results are mixed up in the discussion section.
Moreover, a profound language editing is necessary. Wrong numbering of tables in text.

Abstract:
Methods not cleary described. Though 17 patients were included into the study only six patients received a second MRI. The data of the six patients and not 17 are the main results in this study and might be misleading. As mentioned above mixing values from measurements or signal changes from 3T and 1.5 T MRI inter-individually and intra-individually does not show valid results.

> *Thank you for this important comment. We changed the abstract accordingly to eliminate any misleading confusions. We emphasized that only six patients were included to the artifact reduction measurements, and we described more precisely that different MRI field strengths were applied.*

P1 line 28: wrong order of units. Correct: 0.04 mm (CI: 0.03-0.05).
P1 line 31: wording: not artifact strength, but artifact extent
P1 line 35: Cohen's kappa>0.9. Please give exact value.
Key words: wording: not metallic artifacts, but metal artifact
Abbreviations: Capitalization of the word "X-ray" please!

The wording mistakes were corrected. The exact Cohen's kappa value of 0.88 was indicated.

Main Document
P5 line 16: bone instead of bony
P5 line 22: add "and thus, implant-related…."
P5 line 39: Split it up in two sentences: ….implants. However,
P5 line 45: Please use no space or non-breaking space between end of sentence and citation. Then, there would not be a line break between those.
P5 line 54: "unique" might be exaggerated.
P5 line 56: Wording: it is not "lesser" density. To be correct: "reduced attenuation of X-rays"
P6 line 20: Syntax: Thus, this study
P6 line 33: what are the exclusion criteria. Why were 31 patients out of 48 patients excluded?
P6 line 58: wrong numbering of tables.
P7 line 9: Wording: It is not a 1.5 Tesla. It is 1.5 T MRI or Magnetic field strength.
Table 1: What is the time interval between operation and 1st MRI, 2nd MRI, 3rd MRI?

The time of MRI study is given in Table 1. First MRI is the time between the initial operation and the MRI, and similarly the 2nd and the 3rd MRI is also the time interval between the initial operation and the timing of MRI study.
The mean time interval between operation and MRI (in months) was added to Table 3. All above-mentioned wording mistakes were corrected.

P8 line 33: wording: better or something similar: Patients were referred to MRI imaging at different time points according clinical presentation.
P8 line 39: non-breakig space between 3.0 and T MRI Ingenia.
P9 line 59: Abbreviation COL not described
P10 line 3: Abbreviation ROW not described
P10 line 14: As described above it is not measurements of artifact, but area of signal loss.
P10 line 18: perpendicular measurements only applicable on images in coronar and sagittal orientation. Perpendicular measurements to the screw long axis on axial images not possible as the axial images are oriented to the ankle joint not axial to the screw.
P10 line 25: years of experience?

All above-mentioned wording mistakes were corrected. Especially the issue of artifacts and the signal-loss area was addressed.

Results:
Missing information of qualitative results. How many patients had bone marrow edema, soft tissue changes etc. What is the mean time interval between operation and imaging? This is quite important to evaluate postoperative changes.
P10 line 58: non-breaking space between 2.1 and mm.

Thank you for the important comment. Table 4 with a more detailed description of qualitative findings was added to the results section. The mean time interval between operation and MRI exams (in months) was added to Table 3.

Invalid quantitative results as comparing measurements from 1.5T and 3.0 T MRI images.

This is a very reasonable comment. We fully agree that it is difficult to compare metal artifacts in MR-images acquired in scan units of different field strength. This circumstance is attributed to the retrospective study design and the lack of radiological data with Mg implants in general due to the innovativeness of the material.
The main reason why we nevertheless performed a quantitative analysis is the fact, that some of the second and the third postoperative MRIs (5 cases in total) were performed on a 3T MRI unit, but not 1.5T like the first postoperative

MRIs. None of the patients got a 3T MRI as first follow-up and a 1.5T for second or third follow-up control. Though metal artifacts are expected to be greater in a 3T MRI unit compared to 1.5T MRI unit, we measured a smaller artifact extent in the follow-up (3T) MRI, compared to the first postoperative MRI (1.5T). Therefore, we decided to assemble our results and assume that our measurement are valid despite of the different field strengths and visualize the degradation process of the screw.

We re-evaluated and re-arranged the results section under consideration of your comment. The measurements were given for 1.5T and 3T MRI separately (Table 3). In addition, we tried to address this issue in more detail in the discussion section.

Discussion:
Extensive and good discussion of the topic.
P12 Line 20: What is the clinical impact.
P12 line 54: particularly "with" water
P12 line 56: switch of tempus.
P14 line 20: when exactly did the qualitative changes disappeared. It is not described in the result section.

Table 4 with a description of different findings and the corresponding time intervals was added to the results section.
We did not observe any correlation between clinical symptoms and the reported findings.
The wording mistakes were corrected.

Table legends:
Where are table 1-3 if the tables start with table 4?
P20 line 18: wording: not scanning parameters, but acquisition parameters

Table legends and numbers were changed accordingly.

Figure legends
P21 line 8: wording: not "appearance" but maybe: Image of a MAGNEZIX R compression with 3.2 mm diameter
P21 line 18: not artifact sizes! Artifact extent.
P21 line 31: wording: different MRI..different time points...different patients.
Figures:
Figure 3: Please more extensive description.
Figure 4: What does the images show. Please describe e.g., "Reduced extent of artifacts in image a in comparison to image b"....
Figure 5: what is seen on these beautiful images. Please describe findings in the figure caption

Figure legends / captions were changed accordingly.

We hope the revision improved the manuscript and that in your opinion it is now appropriate for publication.

Response to reviewer 2

Dear Reviewer 2, *thank you for the time and effort spent on reviewing our manuscript. All of your remarks were considered and included into the revised version. All changes are highlighted in green.*
Point-by-point revision

Comments to the Author
Dear authors,
Thank you very much for this interesting work. The questioning, argumentation and presentation of your results are well done.
Introduction: Can you clarify, whether there is a recommendation (guideline) for implant removal in adults? For children, there is such a guideline (DGOUC) recommending the implant removal.

Dear reviewer, there is no universally accepted guideline or an algorithm for routine implant removal after orthopedic surgery. However, if there are symptoms such as irritation, signs of infection, skin problems and similar complaints, the implants are frequently removed. We have added more detailed explanations to the introduction section.

In Table 1: Can you insert the reason for the MRI for each case?. You have to clarify, that the patient was bone healthy and had no signs of osteomyelitis ...

The reason for MRI examination was ankle pain and monitoring the healing process of the mosaicplasty procedure, and imaging of the cartilage. None of the patients had sings of infection or osteomyelitis.
MRI is not a routine postoperative imaging modality for mosaicplasty in the authors' institution; that is why only six patients who had consequent MRI were included.

We hope the revision improved the manuscript and that in your opinion it is now appropriate for publication.

Reviewers' Comments to Revised Submission

Reviewer 2: annomymous

Date received: 01-Oct-2021
Reviewer recommendation: **Accept in present form**
Reviewer overall scoring: **High**

Assessment Form scores: 5 = High/Yes; 3 = Medium/Adequate; 1 = Low

Is the subject area appropriate for the journal		4			
Does the title clearly reflect the paper's content?	5				
Does the abstract clearly reflect the paper's content	5				
Do the keywords clearly reflect the paper's content?	5				
Does the introduction present the problem clearly?		4			
Are the results/ conclusions justified?		4			
How comprehensive and up-to-date is the subject matter presented?	5				
How adequate is the data presentation?	5				
Are units and terminology used correctly?	5				
Is the number of cases adequate?		4			
Are the experimental methods/ clinical studies adequate?		4			
Is the length appropriate in relation to the content?		4			
Does the reader get new insights from the article?	5				
Please rate the practical significance.	5				
Please rate the accuracy of methods.		4			
Please rate the statistical evaluation and quality control.		4			
Please rate the appropriateness of the figures and tables.	5				
Please rate the appropriateness of the references.	5				
Please evaluate the writing style and use of language.		4			
Please judge the overall scientific quality of the manuscript.		4			
Are the methods used worthy of reproduction in greater deal?	Yes				

Would you be willing to review a revision of this manuscript?	Yes

Comments to author: I recommend to accept this paper in the present form..

Comments by the Editor-in-Chief to Revised Submission

Since the second reviewer rated the revised manuscript "5", I feel that we should accept the revised manuscript, although reviewer 1 wants another revision. Since he is not willing to re-review the manuscript, I tend to accept the paper in this revised version.

Comments by the Editorial Office to the Editor-in-Chief Decision

Reviewer 1 was unavailable for the evaluation of the revised manuscript. Since reviewer 2 and the EiC confirmed that all initial comments were addressed by the others in the revision, the decision was finally approved.
